# Radiation-induced accelerated aging of the brain vasculature in young adult survivors of childhood brain tumors

**DOI:** 10.1093/nop/npaa002

**Published:** 2020-02-07

**Authors:** Tiina Maria Remes, Maria Helena Suo-Palosaari, Päivi K T Koskenkorva, Anna K Sutela, Sanna-Maria Toiviainen-Salo, Pekka M Arikoski, Mikko O Arola, Vesa-Pekka Heikkilä, Mika Kapanen, Päivi Maria Lähteenmäki, Tuula R I Lönnqvist, Hannele Niiniviita, Tytti M-L Pokka, Liisa Porra, V Pekka Riikonen, Jan Seppälä, Kirsti H Sirkiä, Antti Vanhanen, Heikki M J Rantala, Arja H Harila-Saari, Marja K Ojaniemi

**Affiliations:** 1 Department of Pediatrics and Adolescence, PEDEGO Research Unit and Medical Research Center, Oulu University Hospital, and University of Oulu, Oulu, Finland; 2 Department of Diagnostic Radiology, Oulu University Hospital, and University of Oulu, Research Unit of Medical Imaging, Physics, and Technology, Faculty of Medicine, University of Oulu, and Medical Research Center Oulu, University of Oulu, Oulu, Finland; 3 Department of Clinical Radiology, Kuopio University Hospital, Kuopio, Finland; 4 Department of Pediatric Radiology, HUS Medical Imaging Center, Radiology, University of Helsinki, and Helsinki University Hospital, Helsinki, Finland; 5 Department of Pediatrics and Adolescence, Kuopio University Hospital, University of Eastern Finland, Kuopio, Finland; 6 Department of Pediatrics, Tampere University Hospital, and University of Tampere, Tampere, Finland; 7 Department of Oncology and Radiotherapy, Oulu University Hospital, Oulu, Finland; 8 Department of Oncology and Department of Medical Physics, Tampere University Hospital, Tampere, Finland; 9 Department of Pediatrics and Adolescent Medicine, Turku University Hospital, and Turku University, Turku, Finland; 10 Department of Child Neurology, Children’s Hospital, University of Helsinki, and Helsinki University Hospital, Helsinki, Finland; 11 Department of Medical Physics, Division of Medical Imaging, Turku University Hospital, Turku, Finland; 12 Department of Oncology, University of Helsinki and Helsinki University Hospital, Helsinki, Finland; 13 Center of Oncology, Kuopio University Hospital, Kuopio, Finland; 14 Department of Pediatrics and Adolescence, Helsinki University, and Helsinki University Hospital, Helsinki, Finland; 15 Uppsala University, Department of Women’s and Children’s Health, Akademiska sjukhuset, Uppsala, Sweden

**Keywords:** brain tumor, cerebrovascular disease, radiotherapy, stroke, survivor

## Abstract

**Background:**

Cranial radiotherapy may damage the cerebral vasculature. The aim of this study was to understand the prevalence and risk factors of cerebrovascular disease (CVD) and white matter hyperintensities (WMHs) in childhood brain tumors (CBT) survivors treated with radiotherapy.

**Methods:**

Seventy CBT survivors who received radiotherapy were enrolled in a cross-sectional study at a median 20 years after radiotherapy cessation. The prevalence of and risk factors for CVD were investigated using MRI, MRA, and laboratory testing. Tumors, their treatment, and stroke-related data were retrieved from patients’ files.

**Results:**

Forty-four individuals (63%) had CVD at a median age of 27 years (range, 16-43 years). The prevalence rates at 20 years for CVD, small-vessel disease, and large-vessel disease were 52%, 38%, and 16%, respectively. Ischemic infarcts were diagnosed in 6 survivors, and cerebral hemorrhage in 2. Lacunar infarcts were present in 7, periventricular or deep WMHs in 34 (49%), and mineralizing microangiopathy in 21 (30%) survivors. Multiple pathologies were detected in 44% of the participants, and most lesions were located in a high-dose radiation area. Higher blood pressure was associated with CVD and a presence of WMHs. Higher cholesterol levels increased the risk of ischemic infarcts and WMHs, and lower levels of high-density lipoprotein and higher waist circumference increased the risk of lacunar infarcts.

**Conclusions:**

Treating CBTs with radiotherapy increases the risk of early CVD and WMHs in young adult survivors. These results suggest an urgent need for investigating CVD prevention in CBT patients.

The risk of early cerebrovascular disease (CVD) is greater among childhood brain tumor (CBT) survivors treated with radiotherapy than among the general population and among CBT survivors who have not been treated with radiotherapy.^1–7^ The clinical consequences of CVD include cognitive impairment, strokes, and increased mortality.^8–13^ Clinical symptoms of CVD may appear several years after the development of vascular pathology.^10,12,14^ CVD poses a major challenge to health-care systems and has a high societal cost.^9–12,15,16^

Knowledge about CVD in survivors of CBTs treated with radiotherapy is based on reports of cavernomas, microbleeds, and large-vessel vasculopathy visualized using magnetic resonance imaging (MRI).^6,13,17–19^ Compared with healthy populations, survivors of CBTs treated with radiotherapy have up to a 100-fold increased risk of stroke.^2–4,7^ However, to date, knowledge about increased stroke risk has been derived from registr**y** data or self-reports.^2–4,7^ The overall picture of CVD after irradiation is not well understood.

CVD may manifest as small-vessel disease and large-vessel disease.^10^ Mineralizing microangiopathy has been reported after cranial irradiation, but is not included in the definition criteria of small-vessel disease in the general population.^1,20–22^ The imaging findings described in CBT survivors that are related to small-vessel disease include microbleeds, cavernomas, and lacunar infarcts.^5,6,13,17,22^ White matter lesions (WMLs) are classified as a small-vessel disease in the general population, but in CBT survivors, the etiology is multifactorial.^22,23^ Large-vessel disease includes ischemic infarcts, large-vessel vasculopathy, and transient ischemic attacks (TIAs).^2,18,19^

This study aimed to understand the prevalence and risk factors for CVD, and white matter hyperintensities (WMHs) by performing brain MRI and MRA on CBT survivors who were treated with radiotherapy.

## Methods

### Study Population

Consecutive CBT survivors who were diagnosed between 1970 and 2008 and treated with radiotherapy (n = 127) were identified from the registers at 5 university hospitals in Oulu, Kuopio, Turku, Tampere, and Helsinki, where all CBTs were treated in Finland. An invitation letter was sent to all included CBT survivors to participate in a study of late complications. The inclusion criteria were patients who (i) were diagnosed at younger than age 16 years, (ii) received radiotherapy as part of their treatment, (iii) were age 16 years or older at the time of the study, (iv) had undergone 5 or more years of follow-up since therapy cessation, and (iv) had no known progressive brain tumors.

All participants were treated with conventional radiotherapy (n = 70). Patients’ records were reviewed regarding the treatment of the primary tumor and occurence of stroke. This is a cross-sectional study in which participants were examined clinically using brain MRI and MRA, and their blood samples were analyzed for the presence of atherosclerotic risk factors during a visit to one of the study centers. Family history of stroke or myocardial infarction at age younger than 50 years was gathered by a questionnaire. Baseline characteristics of participants and nonparticipants are shown in Table 1.

**Table 1:** Baseline Data of Patients and Tumors, and Tumor Treatment Characteristics

	Participants (n = 70)	Nonparticipants (n = 57)	*P*
Sex (males), No. (%)	45 (64)	29 (51)	.150^a^
Age at diagnosis, median (range), y	8.3 (1.1-15.7)	8.5 (0.1-15.7)	.711^b^
Age at radiotherapy, median (range), y	8.5 (1.5-15.9)	8.8 (0.2-15.8)	.604^b^
Age at follow-up visit, median (range), y	27.2 (16.2-43.8)	28.8 (17.8-49.7)	.386^b^
Follow-up time, median (range), y	20.7 (5.0-33.1)	20.9 (6.6-45.1)	.278^b^
Tumor location, n (%)			.723^a^
Infratentorial	37 (53)	28 (49)	
Supratentorial	33 (47)	29 (51)	
Tumor histology, No. (%)			.741^a^
Glial cell tumor	24 (34)	17 (30)	
Embryonal tumor	23 (33)	19 (33)	
Ependymoma	8 (11)	8 (11)	
Germ cell tumor	6 (9)	5 (7)	
Tumor of sellar region^c^	3 (4)	0 (0)	
Other	2 (3)	3 (4)	
No histology	4 (6)	5 (7)	
Total dose of radiotherapy, median (range)	52.6 (30.0-65.4)	53.6 (16.0-72.0)	.311^b^
Radiotherapy, No. (%)^d^			.086^a^
Local	37 (53)	19 (36)	
Craniospinal	30 (43)	27 (51)	
Whole-brain	(4)	5 (9)	
Stereotactic	0 (0)	2 (4)	
Chemotherapy, No. (%)	45 (64)	38 (66)	.709^a^
Ventriculoperitoneal shunt, No. (%)	41 (59)	37 (66)	.461^a^
Surgery, No. (%)			.829^a^
Total resection	29 (41)	19 (33)	
Partial resection	30 (43)	28 (49)	
Biopsy	8 (12)	7 (12)	
No surgery	3 (4)	3 (6)	
Reoperation, No. (%)	19 (27)	12 (21)	.411

^a^Chi-square exact test.

^b^Mann-Whitney U test.

^c^Sellar tumors included adenomas and craniopharyngeomas.

^d^In 4 nonparticipants the exact mode of radiotherapy could not be confirmed from the patient files.

### Imaging

Brain MRI and MRA were performed using a Magnetom Espree 1.5T scanner (Siemens Healthcare GmbH) in Oulu, an Ingenia 1.5T scanner (Philips Healthcare) in Turku, and Avanto 1.5T scanners (Siemens GmbH) in Helsinki, Kuopio, and Tampere. The brain MRI protocol included the following pulse sequences: T1-weighted spin-echo (SE) sagittal, T2-weighted SE axial, T2-weighted fluid-attenuated inversion recovery axial, T1-weighted 3-dimensional inversion recovery SE coronal, axial diffusion-weighted imaging (DWI), and 3-dimensional time-of-flight MRA. After administering gadolinium contrast agent (0.2 mL/kg) (Dotarem; Guerbet), T1-weighted SE axial and T1-weighted SE coronal sequences were conducted.

Viewing applications for diagnostic radiology, which comprised picture archiving and communication systems or digital imaging and communications in medicine, were used to evaluate the MRI scans, namely, neaView (Neagen) in Oulu, Sectra Workstation IDS7 version 19.1.10.3584 (Sectra AB) in Kuopio, and Agfa Impax version 6.6.1.5551 2017 (Agfa Healthcare N.V.) in Helsinki. Radiologists from 3 hospitals evaluated the MRI scans, all of which were reevaluated by M.S.-P. 

### Cerebrovascular Disease Classification Criteria

CVD was defined as a history of cerebral hemorrhage or TIA, which was obtained from reviews of patients’ files or any vascular lesion, excluding WMHs, detected following brain MRI.

Large-vessel disease was diagnosed if the participants had signs of previous ischemic infarct on MRI, or experienced a TIA or presence of large-vessel vasculopathy. Large-vessel vasculopathy was defined as any pathology of the cerebral vessels on MRA. Ischemic infarct was defined according to American Heart and Stroke Associations criteria of brain cell death attributable to ischemia, based on imaging evidence of cerebral focal ischemic injury in a defined vascular distribution.^24^ The definition of TIA according to the American Academy of Neurology was used in the present study (transient episode of neurological dysfunction caused by focal brain and spinal cord or retinal ischemia without acute infarction).^25^ From the clinical files, the etiology and the date of TIAs and ischemic infarcts were determined.

Small-vessel disease was defined as the presence of lacunar infarcts or focal hemosiderin deposits (FHDs).^22^ Given the direct effects of radiotherapy and chemotherapy on the brain, WMHs were not considered signs of small-vessel disease in our cohort.^22,23,26^ Perivascular spaces were excluded in the analysis because of the controversy as to whether they should be considered lesions and the poor understanding of the mechanism underlying enlarged perivascular spaces.^22^

Lacunar infarcts were defined according to international consensus as round or ovoid, subcortical, 3-mm to 15-mm diameter fluid-filled cavities that were consistent with previous acute small subcortical infarcts or hemorrhages in the territory of one perforating arteriole.^22^ International criteria were used to diagnose cerebral microbleeds that were defined as small areas, generally 2 to 5 mm in diameter, but sometimes up to 10 mm in diameter, of signal voids with associated blooming artifacts seen on gradient-echo MRI sequences that were sensitive to susceptibility effects.^22^ In this study, FHDs were noted within low B-value DWI sequences in the absence of susceptibility-weighted imaging or T2* sequences.

**Fig. 1:**
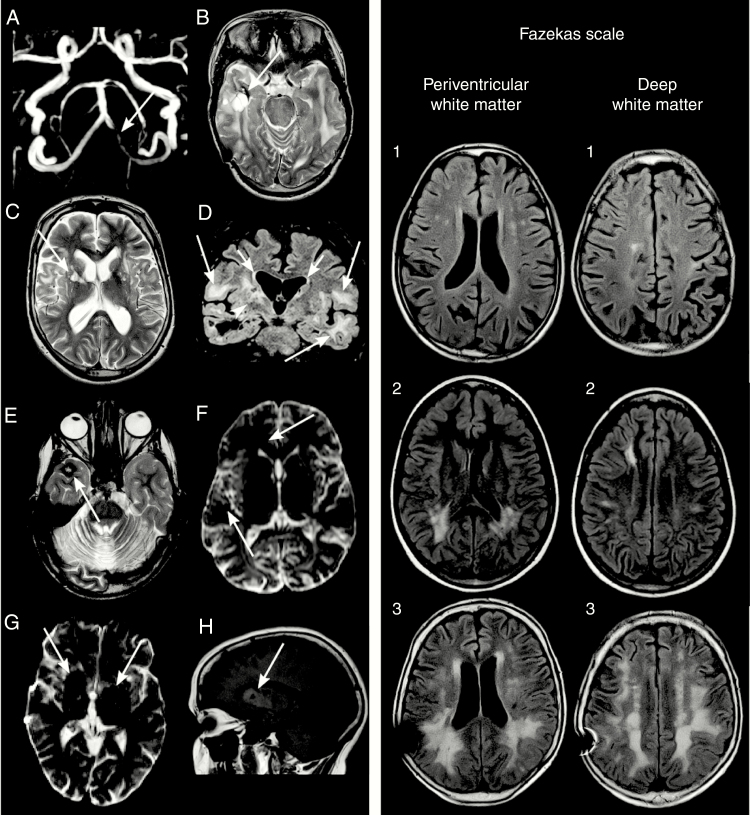
Panel 1, A to D, A, arrow, A 27-year-old male patient had 50% stenosis of the intracranial left vertebral artery on MRA after a follow-up of 21 years. T2-weighted axial images show a B, arrow, 3-cm cavity of hyperintense fluid with a surrounding hypointense hemosiderin rim and septations located in the right temporal lobe indicative of radiation necrosis and a C, arrow, hyperintense lacunar infarct on the right basal ganglia. D, A T2 fluid-attenuated inversion recovery coronal image demonstrated a large confluent of deep white matter (WM) (Fazekas grade 3, long arrows) and moderate periventricular (Fazekas grade 2, short arrows) hyperintensities, suggesting severe vasculopathy. D, arrowhead, The right basal ganglia were surrounded by a T2-hyperintense area, indicating vasculopathy. Panel 1, E to F, E, arrow, A 24-year-old male patient had a cavernoma in the right temporal lobe defined by the presence of a typical hyperintense nidus and a dark rim of hemosiderin on a T2-weighted axial image with no surrounding cerebral edema. F, arrows, On diffusion-weighted imaging, several small, rounded hypointense microbleeds were detected in the right cerebral hemisphere. MRI was performed 17 years after radiotherapy. Panel 1, G to H, G, arrows, A 31-year-old female patient had bilateral hypointense lesions on the basal ganglia on diffusion-weighted imaging that showed a heterogeneous signal on H, arrow, T1-weighted sagittal images typical of calcifications. The follow-up time of the female patient was 23 years. Panel 2 demonstrates representative T2 fluid-attenuated inversion recovery images of mild (grade 1), moderate (grade 2), and severe (grade 3) cerebral WM hyperintensities classified using the Fazekas scale (Table 1).^26^ A 41-year-old male patient showed multiple, small, punctate deep WM Fazekas grade 1 hyperintensities after a 23-year follow-up. A 19-year-old female patient demonstrated after a follow-up time of 11 years several periventricular and deep WM Fazekas grade 2 hyperintensities. A 43-year-old male patient showed large confluent periventricular and deep WM Fazekas grade 3 hyperintensities after a follow-time of 26 years.

We used the international definition of WMH, that is, signal abnormalities of variable sizes in the white matter that show hyperintensity on T2-weighted images without cavitations.^22^ WMHs were classified using the Fazekas scale based on their distributions and sizes of the periventricular and deep WMHs (Table 2; Fig. 1).^27^

**Table 2:** Cerebrovascular Disease, Cerebrovascular Findings, and Fazekas Scale Grades Among Study Participants (n = 70)

	No. (%)
Cerebrovascular disease	45 (63)
Large-vessel disease	13 (19)
Ischemic stroke	6 (9)
Transient ischemic attack	2 (3)
Large-vessel vasculopathy	6 (9)
Small-vessel disease	27 (39)
Lacunar infarcts	7 (10)
Focal hemosiderin deposits	23 (33)
Cerebral hemorrhage	2 (3)
Mineralizing microangiopathy	21 (30)
White matter hyperintensities	34 (49)
Fazekas scale^a^	
Periventricular hyperintensities	
0 (Absent)	50 (72)
1 (“caps” or pencil-thin lining)	12 (17)
2 (smooth “halo”)	7 (10)
3 (irregular PVH extending into the deep white matter)	1 (1)
Deep white matter hyperintensity	
0 (absent)	45 (64)
1 (punctate foci)	13 (19)
2 (confluence of foci beginning)	9 (13)
3 (large confluent areas)	3 (4)
Total amount of different pathologies per person^b^	
0	16 (23)
1	23 (33)
2	17 (24)
3	9 (13)
4	5 (7)

Abbreviation: PVH, periventricular hyperintensity.

^a^Fazekas scale, according to Fazekas et al.^26^

^b^The amount has been calculated based on the number of different vascular pathologies or white matter lesions detected per person.

Mineralizing microangiopathy was diagnosed as signs of calcifications on MRI. The definition of the American Stroke Association was used to diagnose cerebral hemorrhage as a nontraumatic focal collection of blood within the brain parenchyma or ventricular system.^24^ Although subdural hematomas may occur spontaneously, they are not included in the definition of stroke.^24^ We used Common Terminology Criteria for Adverse Events (CTCAE) classification version 5.0 for reporting the severity of strokes, TIAs, and traumatic and subdural hemorrhages.^28^ In this classification, grade 1 is used to describe mild severity of an adverse event, 2 for moderate severity, 3 for severe or medically significant but not immediately life-threatening, 4 for life-threatening consequences, and 5 for death.^28^

### Laboratory Analyses

Laboratory samples were collected after overnight fasting, and all analyses were performed in the Nordlab at Oulu University Hospital. Plasma glucose, cholesterol, high-density lipoprotein (HDL), low-density lipoprotein, triglyceride, and glycosylated hemoglobin A_1c_ levels were measured and analyzed using a clinical chemistry system (Advia 1800; Siemens Healthcare GmbH). Serum insulin levels were analyzed using a chemiluminescent immunoassay (Advia Centaur XP; Siemens Healthcare GmbH). One participant’s blood samples could not be collected and another individual had not fasted, so these samples were excluded from the analyses. Homeostatic model assessment of insulin resistance index was calculated using fasting glucose and insulin levels for quantifying insulin resistance.^29^

### Radiation Dose Distribution Analyses

The majority of the patients were treated in the 1980s and 1990s using 2-dimensional treatment planning techniques. The medical physicists analyzed dose distributions using patients’ charts, treatment plans, and radiation field images to determine the radiation doses. Nine patients’ radiation field images and treatment plans were not available; among them, 4 patients were treated with local radiotherapy and 5 with whole-brain radiotherapy.

### Statistical Analyses

Differences between the median values of 2 independent groups were tested using the Mann-Whitney U test. Differences between the median values of 4 independent groups were tested using the Kruskal-Wallis test. The chi-square exact test was used to compare the distribution of categorical variables relative to categorical variables in other groups. For CVDs, only the time before the event occurred was known and interval-censored survival analysis with the EMICM algorithm was used to calculate the cumulative prevalence. Logistic regression analysis was used to calculate the odds ratios (ORs) for the atherosclerotic risk factors and MRI markers; the results are presented as ORs and their 95% CIs. Statistical analyses were performed using IBM SPSS software, version 25 for Windows (IBM Corp) and SAS 9.4 (SAS institute Inc). Graphs were produced using OriginPro 2018 software (OriginLab).

### Ethics

Written informed consent was obtained from all enrolled participants or their legal guardians. The study was approved by the institutional review boards at Oulu, Kuopio, Turku, Tampere, and Helsinki University Hospitals, Finland. The research was conducted according to the principles of the Declaration of Helsinki.

## Results

### Patients’ Characteristics

Of the 127 initially eligible individuals, 70 (45 male) participated in the study. Forty people declined to participate and 13 were lost to follow-up. Two participants did not undergo craniospinal MRI because of vagus nerve stimulator (n *=* 1) and claustrophobia (n = 1). Two survivors treated with stereotactic radiotherapy were excluded from the analysis.

The median age of the participants at the time of imaging was 27.1 years (range, 16.2-43.8 years), and their median age at tumor diagnosis was 8.3 years (range, 1.1-15.7 years). The median interval from the end of the radiotherapy course to imaging was 20.7 years (range, 5.0-33.1 years). Radiotherapy was given between 1980 and 2007. One patient was reradiated year after the first treatment, so that the total dose of radiation was 51.0 Gy. Type 1 neurofibromatosis was diagnosed in one participant. Radiotherapy for glial cell tumors has been reduced over the years; after 2000, only one glial tumor was treated with radiotherapy in this cohort. The median follow-up time was shorter for survivors treated with whole-brain radiotherapy than for those treated with local radiotherapy (16.5 years [5.0-21.2 years] vs 21.5 years [8.2-33.1 years]; *P* = .003).

### Cerebrovascular Disease

**Fig. 2:**
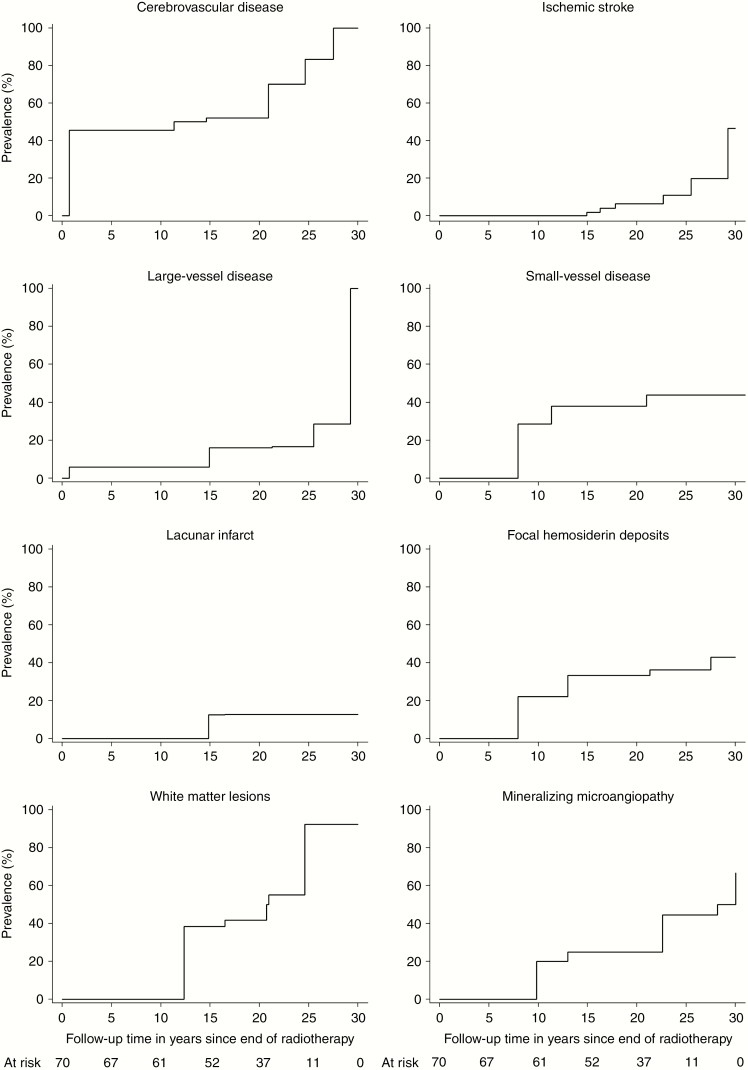
Prevalence of cerebrovascular disease in a cohort of childhood brain tumor survivors studied in a cross-sectional setting. Follow-up intervals from the cessation of radiation therapy to stroke or MRI for cerebrovascular disease, large-vessel disease, and ischemic infarcts. Follow-up interval from the cessation of radiation therapy to MRI for small-vessel disease, lacunar infarcts, focal hemosiderin deposits, mineralizing microangiopathy, and white matter lesions. TIA, transient ischemic attack.

CVD was diagnosed in 63% of the participants (Table 2). The cumulative prevalence of CVD at 20 years of follow-up was 52% (95% CI, 39%-66%, Fig. 2). A total of 44% of the participants had coexisting imaging findings in the brain (Table 2).

### Large-Vessel Disease

Large-vessel disease, which was diagnosed based on the presence of ischemic infarct, TIAs, or large-vessel vasculopathy, was present in 19% of the participants (Table 2). The cumulative prevalence of large-vessel disease at 20 years of follow-up was 16% (95% CI, 9%-28%, Fig. 2). Overall, 8 individuals had either TIAs (n = 2) or late-occurring ischemic infarcts (n = 6, Table 2). One perioperative ischemic infarct was excluded from all analyses. In the brain MRI, ischemic infarcts were in the occipital (n = 3), frontal (n = 1), and temporal lobes (n = 1) as well as the temporoparietal area (n = 1), basal ganglia (n = 1), and thalamic area (n = 1). Two ischemic infarcts were detected in 2 individuals; recurrence was seen in 1 participant. In the individual with recurrent stroke, the first ischemic infarct was found in the left occipital lobe, and the recurrence was in the left temporal lobe. No vascular pathology was found on the MRA. Ischemic infarcts were classified as CTCAE grade 1 (n = 3), grade 2 (n = 4), and grade 3 (n *=* *1*). CTCAE classifications for the 2 TIAs were grade 1 and 2. The participant with recurrent ischemic infarcts reported using clopidogrel as an antiplatelet medication at the time of the study; the other individuals with ischemic infarcts or TIAs did not report using antiplatelet therapy. The cumulative prevalence of ischemic infarcts at 20 years of follow-up was 6% (95% CI, 3%-15%, Fig. 2).

MRA detected large-vessel vasculopathy in 6 participants, including stenotic caliber changes in the right middle cerebral artery (MCA, n = 1), vasculopathy in the right MCA with ischemic infarct in the right basal ganglia on MRI (n = 1), and stenoses in the left posterior cerebral artery (n = 1) and the left vertebral artery with a lacunar infarct in the right basal ganglia on MRI (n = 1, Fig. 1, 1A). In one individual, neither the right MCA nor the anterior cerebral arteries could be visualized. One patient had undergone surgery for a left frontal arteriovenous malformation after radiotherapy.

### Small-Vessel Disease

Small-vessel disease, diagnosed as the presence of lacunar infarcts or FHDs, was found in 39% of the participants (Table 2). The cumulative prevalence of small-vessel disease at 20 years of follow-up was 38% (95% CI, 27%-51%, Fig. 2). Five (7%) individuals had large-vessel disease and small-vessel disease, 22 (31%) were diagnosed with small-vessel disease only, and 8 (11%) were diagnosed with large-vessel disease only.

Lacunar infarcts (Fig. 1 C) were present in 10% of the participants (Table 2), and 2 individuals had multiple lacunar infarcts. Lacunar infarcts (n = 11) were situated in the capsula externa (n = 5), nucleus lentiformis (n = 4), corona radiata (n = 1), and thalamus (n = 1). Lacunar and ischemic infarct coexisted in one individual. A representative image is shown in Fig. 1 C. The cumulative prevalence of lacunar infarcts at 20 years of follow-up was 13% (95% CI, 7%-23%, Fig. 2).

FHDs were found in 33% of the participants (Table 2). Cavernomas (Fig. 1E) were detected in 11 individuals and microbleeds (Fig. 1 F) in 16, with multiple occurrences in 5 and 4 participants, respectively. The cumulative prevalence of FHDs at 20 years of follow-up was 33% (95% CI, 23%-46%, Fig. 2).

### Cerebral Hemorrhage

Cerebral hemorrhage was confirmed in 2 (3%) individuals; both were intracerebral hemorrhages (Table 2). All hemorrhages occurred more than 1 year after radiotherapy. In addition, 3 traumatic and 2 subdural hemorrhages had been diagnosed. Traumatic cerebral hemorrhages included subdural hemorrhages (n = 2) and contusion hematomas (n = 1). Only one participant with cerebral hemorrhage did not have any other signs of CVD. An ischemic infarct and a subdural hemorrhage coexisted in one individual. CTCAE classification for 2 cerebral hemorrhages were grade 1, as well as for all traumatic and subdural hemorrhages.

### Mineralizing Microangiopathy

Mineralizing microangiopathy was detected in 30% of the participants, with a cumulative prevalence at 20 years of follow-up of 25% (95% CI, 16%-39%, Table 2 and Fig. 2). Figure 1 G and H, shows a scan with calcifications characteristic of mineralizing microangiopathy.

### Telangiectasia

One participant had a left thalamic slightly T1- and T2-hyperintense lesion, which revealed a brush-like enhancement with gadolinium contrast media that was indicative of telangiectasia.

### White Matter Hyperintensities

WMHs quantified by the Fazekas scale were observed in 49% of the participants (Table 2). Periventricular hyperintensities and deep WMHs were detected in 28% and 36% of the individuals, respectively (Table 2). Fig. 1 panel 2, shows representative MRI scans of WMHs, and Table 2 presents the distribution of the WMHs according to the Fazekas scale. WMHs were seen in survivors followed for more than 10 years. The cumulative prevalence at 20 years of follow-up was 42% (95% CI, 29%-57%, Fig. 2).

### Clinical Symptoms of Cerebrovascular Disease

Of the 70 survivors, epilepsy was diagnosed in 22 (31%). A total of 10 (14%) survivors reported having regular headaches and 21 (30%) other symptoms, such as psychiatric symptoms in 10 (15%) survivors, memory problems in 6 (9%), and vision problems in 5 (7%). Epilepsy was associated with large-vessel disease (OR, 4.91; 95% CI 1.38-17.49; *P = *.014) and WMHs (OR, 4.44; 95% CI 1.47-13.42; *P* = .008). Epilepsy was not associated with CVD (OR, 1.40; 95% CI 0.48-4.08; *P* = .533), small-vessel disease (OR, 0.87; 95% CI 0.31-2.48; *P = *.797), ischemic infarcts (OR, 5.11; 95% CI 0.86-30.39; *P* = .073), or lacunar infarcts (OR, 1.74; 95% CI 0.35-8.52; *P = *.496). Headache was not associated with CVD (OR, 1.45; 95% CI 0.34-6.18; *P* = .615), large-vessel disease (OR, 0.44; 95% CI 0.05-3.86; *P* = .462), small-vessel disease (OR, 1.07; 95% CI 0.27-4.22; *P* = .920), or WMHs (OR, 1.71; 95% CI 0.44-6.70; *P = *.438). No other symptoms were associated with CVD (OR, 1.31; 95% CI 0.45-3.84; *P = *.622), large-vessel disease (OR, 1.02; 95% CI 0.28-3.77; *P = *.977), small-vessel disease (OR, 0.76; 95% CI 0.26-2.24; *P = *.622), or WMHs (OR, 0.57; 95% CI 0.20-1.61; *P = *.287).

### Cerebrovascular Risk Factors

#### Tumor- and treatment-related risk factors

Tumor- and treatment-related risk factors are shown in Supplemental Tables 1 and 2. Total dose of radiation was not associated with CVD, large-vessel disease, small-vessel disease, or strokes. Age at the follow-up visit was associated with large-vessel disease and WMHs. Ventriculoperitoneal shunt was associated with small-vessel disease; more specifically with FHDs (OR 3.76; 95% CI, 1.20-11.79; *P* = .023). Increasing radiation dose increased the presence of mineralizing microangiopathy (OR, 1.28; 95% CI 1.06-1.54; *P = *.010). Most of the CVD-associated imaging findings (68%) were located in the regions that received radiation doses 40 Gy or greater. A total of 11% of the microbleeds were found outside the radiation fields (Table 3).

**Table 3:** Imaging Findings of Cerebrovascular Disease and Irradiation Doses

	Microbleeds	Cavernomas	Ischemic Infarcts	Lacunar Infarcts	Mineralizing Microangiopathy	All
	**(n = 27)**	**(n = 17)**	**(n = 8)**	**(n = 9)**	**(n = 38)**	**(n = 99)**
Radiation dose, No. (%), Gy						
More than 50	2 (7)	3 (18)	5 (63)	4 (44)	26 (68)	40 (41)
40 to 49.9	9 (33)	8 (47)	2 (25)	4 (44)	4 (11)	27 (27)
30 to 39.9	8 (30)	5 (29)	0 (0)	0 (0)	4 (11)	17 (17)
20 to 29.9	5 (19)	1 (6)	1 (12)	0 (0)	0 (0)	7 (7)
10 to 19.9	0 (0)	0 (0)	0 (0)	1 (12)	2 (5)	3 (3)
1 to 9.9	0 (0)	0 (0)	0 (0)	0 (0)	2 (5)	2 (2)
0	3 (11)	0 (0)	0 (0)	0 (0)	0 (0)	3 (3)
Mean dose (SD)	33.4 (15.0)	42.6 (8.7)	45.3 (8.3)	44.2 (12.3)	45.1 (14.0)	

*n* equals number of MRI findings.

Radiation doses were analyzed as the maximum doses to the lobe of the brain or to the thalamic area, where the imaging finding was located.

Most tumor characteristics and treatment protocols were not associated with Fazekas grade, except deep WMH, which was associated with total radiotherapy dose (*P = *.035) and body mass index (*P* = .011, Supplemental table 3).

### Atherosclerotic Risk Factors

Male patients were more likely to have small-vessel disease than female patients (OR, 8.38; 95% CI, 2.19-32.04; *P* = .002). The CVD risk rose 1.03-fold (*P* = .045); specifically, the large-vessel disease risk increased 1.05-fold (*P* = .021) for every mm Hg increase in systolic blood pressure. Increasing diastolic blood pressure elevated the large-vessel disease risk 1.07-fold (*P* = .027) and the lacunar infarct risk 1.08-fold (*P* = .031). Total cholesterol and low-density lipoprotein levels were associated with the occurrence of ischemic infarct. Lower HDL levels were associated with lacunar infarcts. Participants with WMHs had higher total cholesterol and systolic and diastolic blood pressure (Table 4). Waist circumference was associated with occurrence of lacunar infarcts. Markers of glucose metabolism, diabetes, smoking, or estrogen therapy were not associated with the prevalence of CVD (Supplemental table 4). A total of 17 survivors had a family member diagnosed with stroke or myocardial infarction at age 50 years or younger. Family history had no association with CVD (Supplemental table 4).

**Table 4:** Association of Cerebrovascular Disease, Strokes, White Matter Hyperintensities, and Atherosclerotic Risk Factors

	Systolic BP in mm HG	Diastolic BP in mm HG	Cholesterol in mmol/L	HDL in mmol/L	LDL in mmol/L
	**Mean (SD)**	**Mean (SD)**	**Mean (SD)**	**Mean (SD)**	**Mean (SD)**
Cerebrovascular disease					
Yes^a^	134 (17)^b^	82 (12)^b^	4.8 (1.1)^b^	1.3 (0.4)^b^	3.2 (1.2)^b^
No^c^	125 (15)^d^	79 (11)^d^	4.6 (0.9)	1.4 (0.4)	2.9 (0.9)
OR	1.03	1.03	1.18	0.67	1.31
95% CI	1.00-1.07	0.98-1.07	0.72-1.93	0.20-2.19	0.80-2.13
*P*^*e*^	.045^f^	.284	.509	.503	.284
Small-vessel disease					
Yes^g^	135 (19)^c^	82 (14)^c^	4.8 (1.1)^c^	1.3 (0.3)^c^	3.3 (1.1)^c^
No^h^	128 (15)^i^	79 (10)^i^	4.6 (1.0)^b^	1.4 (0.5)^b^	3.0 (1.1)^b^
OR	1.02	1.02	1.15	0.52	0.81
95% CI	0.99-1.05	0.98-1.07	0.72-1.75	0.15-1.85	0.51-1.29
*P*	.155	.279	.571	.314	.367
Large-vessel disease					
Yes^j^	142 (16)^**k**^	88 (12)^**k**^	5.2 (1.4)^k^	1.4 (0.6)^k^	3.3 (1.6)^k^
No^l^	128 (16)^m^	79 (11)^m^	4.6 (0.9)^n^	1.3 (0.4)^n^	3.1 (1.0)^n^
OR	1.05	1.07	1.68	1.40	1.28
95% CI	1.01-1.10	1.01-1.14	0.94-2.99	0.32-6.05	0.72-2.24
*P*^*e*^	.021^f^	.027^f^	.077	.652	.400
Ischemic infarct					
Yes^o^	135 (15)	82 (11)	5.8 (1.8)^p^	1.2 (0.3)^p^	4.1 (1.7)^p^
No^q^	130 (17)^r^	80 (12)^r^	4.6 (0.9)^s^	1.4 (0.4)^s^	3.1 (1.0)^s^
OR	1.02	1.01	2.41	0.38	2.27
95% CI	0.97-1.07	0.94-1.09	1.09-5.34	0.03-4.96	1.01-5.07
*P*^*e*^	.492	.772	.030^f^	.463	.047^f^
Lacunar infarct					
Yes^t^	142 (21)	90 (14)	4.6 (0.9)^u^	1.0 (0.2)^u^	3.2 (0.7)^u^
No^v^	130 (16)^w^	79 (11)^w^	4.7 (1.1)^x^	1.4 (0.4)^x^	3.1 (1.1)^x^
OR	1.05	1.08	0.89	0.01	1.13
95% CI	0.99-1.10	1.01-1.17	0.38-2.09	0.00-0.45	0.53-2.41
*P*^*e*^	.082	.031^f^	.784	.018^f^	.757
White matter hyperintensities					
Yes^y^	135 (17)^**z**^	85 (11)^**z**^	5.0 (1.2)^v^	1.3 (0.5)^aa^	3.3 (1.2)^aa^
No^ab^	127 (16)^z^	76 (11)^z^	4.4 (0.8)^ab^	1.3 (0.4)^ab^	2.9 (0.9)^ab^
OR	1.03	1.07	1.83	1.04	1.50
95% CI	1.00-1.07	1.02-1.13	1.06-3.16	0.33-3.33	0.92-2.45
*P*^*e*^	.042^f^	.008^f^	.030^f^	.948	.107

Abbreviations: BP, blood pressure; HDL, high-density lipoprotein; LDL, low-density lipoprotein; OR, odds ratio.

^a^n = 44.

^b^n = 41.

^c^n = 26.

^d^n = 25.

^e^Logistic regression analysis.

^f^Significance level equals .05.

^g^n = 27.

^h^n = 43.

^i^n = 40.

^j^n = 13.

^k^n = 12.

^l^n = 57.

^m^n = 54.

^n^n = 55.

^o^n = 6.

^p^n = 5.

^q^n = 64.

^r^n = 60.

^s^n = 62.

^t^n = 7.

^u^n = 6.

^v^n = 63.

^w^n = 59.

^x^n = 61.

^y^n = 34.

^z^n = 33.

^aa^n = 32.

^ab^n = 35.

In the logistic regression analysis of atherosclerotic risk factors, systolic blood pressure was associated with CVD and large-vessel disease. Diastolic blood pressure was associated in the multivariable analysis with lacunar infarcts. In logistic regression analysis of atherosclerotic risk factors, WMHs were associated with diastolic blood pressure. In multivariable analysis, lacunar infarcts were associated with HDL and diastolic blood pressure (Supplemental table 5).

### Association Between MRI Findings and Stroke

No associations were found between WMHs, FHDs, or mineralizing microangiopathy and strokes (see Supplemental Table 2).

## Discussion

Radiotherapy is essential for the treatment of many CBTs, but it causes significant damage to the brain vasculature and can cause early CVD.^1–7,13,17–19,21,30–32^ In this study, most of the long-term CBT survivors who received radiotherapy developed CVD, and the cumulative prevalence of CVD at 20 years of follow-up (52%) was alarmingly high. The rates of ischemic infarct, microbleeds, and lacunar infarct were similar to or higher than those in the general population age 70 years or older, which suggests accelerated aging of the cerebral vasculature.^33–35^ Atherosclerotic risk factors further aggravated the effects of radiotherapy in CBT survivors. CVD and WMLs are known late effects of irradiation, and their relationships with cognitive impairment are recognized.^2–4,6,7,17,20,36^ Generating knowledge about the prevalence of and risk factors for CVD in CBT survivors is crucial for disease prevention.

In Finland, the incidence of ischemic infarcts is 6.6 in the age group of 18 to 34 years and 25.8 in the age group of 35 to 44 years per 100 000 people.^37^ In this study, the cumulative prevalence of ischemic infarcts at 20 years of follow-up was 6%. The cumulative incidence of stroke was 12% at 30 years of follow-up in the Childhood Cancer Survivor Study.^3^ Despite the fact that most cerebral hemorrhages are not classified as strokes, the high prevalence of cerebral hemorrhage shows the fragility of the cerebral vasculature in survivors.^13,24^ The high prevalence in this study might be related to the use of systematic MRI screening.

Moyamoya disease has been frequently reported after cranial radiotherapy, but this was not seen in our cohort.^19,32^ The sensitivity of MRA is not as high as that associated with conventional angiography in relation to detecting Moyamoya disease, but MRA revealed large-vessel vasculopathy in 6 cases.^19^

The high prevalence of small-vessel disease might reflect the greater sensitivity of small vessels to the effects of radiation. The high prevalence of FHDs and the association between microbleeds and cognitive impairment in CBT survivors have been recognized.^6,13,17^ The use of sensitive MRI techniques, such as susceptibility-weighted imaging or T2* sequences, explains the even higher rates of small-vessel disease and microbleeds in some studies.^13,31,38^ Although the number of microbleeds is likely to increase during follow-up both in adult and pediatric brain tumor survivors, some microbleeds may disappear during follow-up.^17,39^

Ischemic infarct risk has been associated with higher total radiation doses, especially those applied to the area surrounding the circle of Willis.^2,3,7,30^ In this study, almost all CVD imaging findings, except for some microbleeds, were located in the radiation field. This suggests that reducing the dose and the size of the radiation field should be beneficial for brain vasculature. Although it has been taught that proton beam radiotherapy could be less harmful than photon radiotherapy because the radiation does not scatter to surrounding tissues, recent studies have shown large-vessel vasculopathy and high incidence of microbleeds after proton beam radiotherapy.^18,40^ However, we did not find a lower rate of CVD in those treated with local radiotherapy, which may be related to fewer glial cell tumors, and thus less local radiotherapy in the survivors with shorter follow-up time. However, even stereotactic radiotherapy reduces cerebral blood flow in the surrounding tissue, which suggests that irradiation may harm the brain vasculature beyond the radiation field.^41^

Atherosclerotic risk factors may further increase the risk of radiation-induced CVD in CBT survivors. Hypertension is a well-known risk factor associated with stroke, and its association with lacunar infarcts has been established in the general population.^42,43^ In this study, higher BP was associated with CVD, large-vessel disease, and lacunar infarcts. A previous study’s findings showed that hypertension was associated with a 4-fold increase in the risk of stroke among CBT survivors.^3^ Higher cholesterol levels were associated with ischemic infarcts, but the mean cholesterol levels were not particularly high. The beneficial effect of antihypertensive medication on stroke risk, even in the high-risk population, is well established.^44,45^ Balancing cholesterol levels in survivors presenting both vascular insufficiency and early atherosclerosis is challenging.^2^ Total cholesterol levels have been inversely associated with hemorrhagic stroke, but directly associated with ischemic strokes in the general population.^46–48^ Future prevention studies for CVD should consider antihypertensive treatment in CBT survivors.

Although the use of antiplatelet therapy for secondary prevention of stroke is well established, it is not currently known how incidental vascular findings should be managed even in the general population.^8,24^ We found a high prevalence of lacunar infarcts, WMHs, and microbleeds, which are all associated with hemorrhagic strokes in the general population.^24^ A high prevalence of cerebral hemorrhagic lesions in CBT survivors suggests vascular fragility. Because survivors of CBT treated with radiotherapy are predisposed both to ischemic and hemorrhagic lesions, treating CVD is particularly challenging in this patient population.

WMHs were relatively common in this study (49%). In the general population, the reported rates are 10% to 21% at around age 64 years and 38% to 88% at age 82 years.^49,50^ WMLs correlate significantly with impaired cognitive function in CBT survivors who received radiotherapy.^20,34^ The associations between WMLs and atherosclerotic risk factors are widely acknowledged within the general population.^8,12^ Similarly to adult cancer patients undergoing whole-brain radiotherapy, higher blood pressure among CBT survivors was associated with a presence of WMHs in this study.^51^ Antihypertensive treatment and lowering blood pressure may even reduce the progression of WML in the general population.^52^

In cancer survivors, the etiology behind periventricular and deep WMH may differ.^53,54^ Radiation-induced and chemotherapy-induced changes in white matter are typically seen in the periventricular area and are thought to result from demyelination, gliosis, edema, and coagulation necrosis.^54^ Deep WMH on MRI may mimic those seen in older patients and those with cerebrovascular risk factors.^53^ In the present study, we could not find significant associations supporting this difference.

This study’s limitations relate to its cross-sectional design and lack of follow-up regarding the development of vascular changes. In addition, patients were treated using different chemotherapy protocols and radiotherapy techniques. However, the total radiation dose administered to the CNS was relatively homogeneous. This study’s strengths are associated with the systematic use of MRI screening on a cohort of consecutive adult CBT survivors who received radiotherapy and the reasonably high participation rate.

In summary, we found an alarmingly high prevalence of CVD as a late complication of cranial irradiation among CBT survivors, and many of the CBT survivors experienced strokes during the follow-up period. Although cranial irradiation remains necessary for the treatment of CBTs, strategies to prevent and treat its late effects on cerebral vasculature are urgently required.

## Supplementary Material

npaa002_suppl_Supplementary_Table_1

npaa002_suppl_Supplementary_Table_2

npaa002_suppl_Supplementary_Table_3

npaa002_suppl_Supplementary_Table_4

npaa002_suppl_Supplementary_Table_5

## References

[1] Morris B, Partap S, Yeom K, et al. Cerebrovascular disease in childhood cancer survivors: a Children’s Oncology Group report. Neurology. 2009;73(22):1906–1913. 19812380 10.1212/WNL.0b013e3181c17ea8 PMC2788797

[2] Campen CJ, Kranick SM, Kasner SE, et al. Cranial irradiation increases risk of stroke in pediatric brain tumor survivors. Stroke. 2012;43(11):3035–3040. 22968468 10.1161/STROKEAHA.112.661561 PMC3492057

[3] Mueller S, Fullerton HJ, Stratton K, et al. Radiation, atherosclerotic risk factors, and stroke risk in survivors of pediatric cancer: a report from the Childhood Cancer Survivor Study. Int J Radiat Oncol Biol Phys. 2013;86(4):649–655. 23680033 10.1016/j.ijrobp.2013.03.034 PMC3696633

[4] Noje C, Cohen K, Jordan LC Hemorrhagic and ischemic stroke in children with cancer. Pediatr Neurol. 2013;49(4):237–242. 23942224 10.1016/j.pediatrneurol.2013.04.009 PMC3783522

[5] Fouladi M, Langston J, Mulhern R, et al. Silent lacunar lesions detected by magnetic resonance imaging of children with brain tumors: a late sequela of therapy. J Clin Oncol. 2000;18(4):824–831. 10673524 10.1200/JCO.2000.18.4.824

[6] Passos J, Nzwalo H, Valente M, et al. Microbleeds and cavernomas after radiotherapy for paediatric primary brain tumours. J Neurol Sci. 2017;372:413–416. 27856004 10.1016/j.jns.2016.11.005

[7] Bowers DC, Liu Y, Leisenring W, et al. Late-occurring stroke among long-term survivors of childhood leukemia and brain tumors: a report from the Childhood Cancer Survivor Study. J Clin Oncol. 2006;24(33):5277–5282. 17088567 10.1200/JCO.2006.07.2884

[8] Debette S, Schilling S, Duperron MG, Larsson SC, Markus HS Clinical significance of magnetic resonance imaging markers of vascular brain injury: a systematic review and meta-analysis. JAMA Neurol. 2019;76(1):81–94. 30422209 10.1001/jamaneurol.2018.3122 PMC6439887

[9] Staals J, Makin SD, Doubal FN, Dennis MS, Wardlaw JM Stroke subtype, vascular risk factors, and total MRI brain small-vessel disease burden. Neurology. 2014;83(14):1228–1234. 25165388 10.1212/WNL.0000000000000837 PMC4180484

[10] Xu X, Hilal S, Collinson SL, et al. Association of magnetic resonance imaging markers of cerebrovascular disease burden and cognition. Stroke. 2015;46(10):2808–2814. 26330446 10.1161/STROKEAHA.115.010700

[11] Wilson D, Charidimou A, Ambler G, et al. Recurrent stroke risk and cerebral microbleed burden in ischemic stroke and TIA: a meta-analysis. Neurology. 2016;87(14):1501–1510. 27590288 10.1212/WNL.0000000000003183 PMC5075978

[12] Debette S, Markus HS The clinical importance of white matter hyperintensities on brain magnetic resonance imaging: systematic review and meta-analysis. BMJ. 2010;341:c3666. 20660506 10.1136/bmj.c3666 PMC2910261

[13] Neu MA, Tanyildizi Y, Wingerter A, et al. Susceptibility-weighted magnetic resonance imaging of cerebrovascular sequelae after radiotherapy for pediatric brain tumors. Radiother Oncol. 2018;127(2):280–286. 29605477 10.1016/j.radonc.2018.03.010

[14] Akoudad S, Ikram MA, Koudstaal PJ, et al. Cerebral microbleeds are associated with the progression of ischemic vascular lesions. Cerebrovasc Dis. 2014;37(5):382–388. 24970709 10.1159/000362590 PMC5291936

[15] Akoudad S, Wolters FJ, Viswanathan A, et al. Association of cerebral microbleeds with cognitive decline and dementia. JAMA Neurol. 2016;73(8):934–943. 27271785 10.1001/jamaneurol.2016.1017 PMC5966721

[16] Jennum P, Iversen HK, Ibsen R, Kjellberg J Cost of stroke: a controlled national study evaluating societal effects on patients and their partners. BMC Health Serv Res. 2015;15:466. 26464109 10.1186/s12913-015-1100-0 PMC4604706

[17] Roddy E, Sear K, Felton E, et al. Presence of cerebral microbleeds is associated with worse executive function in pediatric brain tumor survivors. Neuro Oncol. 2016;18(11):1548–1558. 27540084 10.1093/neuonc/now163 PMC5063522

[18] Kralik SF, Watson GA, Shih CS, Ho CY, Finke W, Buchsbaum J Radiation-induced large vessel cerebral vasculopathy in pediatric patients with brain tumors treated with proton radiation therapy. Int J Radiat Oncol Biol Phys. 2017;99(4):817–824. 28867358 10.1016/j.ijrobp.2017.07.009

[19] Omura M, Aida N, Sekido K, Kakehi M, Matsubara S Large intracranial vessel occlusive vasculopathy after radiation therapy in children: clinical features and usefulness of magnetic resonance imaging. Int J Radiat Oncol Biol Phys. 1997;38(2):241–249. 9226309 10.1016/s0360-3016(97)82497-2

[20] Fouladi M, Chintagumpala M, Laningham FH, et al. White matter lesions detected by magnetic resonance imaging after radiotherapy and high-dose chemotherapy in children with medulloblastoma or primitive neuroectodermal tumor. J Clin Oncol. 2004;22(22):4551–4560. 15542806 10.1200/JCO.2004.03.058

[21] Poussaint TY, Siffert J, Barnes PD, et al. Hemorrhagic vasculopathy after treatment of central nervous system neoplasia in childhood: diagnosis and follow-up. AJNR Am J Neuroradiol. 1995;16(4):693–699. 7611024 PMC8332247

[22] Wardlaw JM, Smith EE, Biessels GJ, et al; STandards for ReportIng Vascular changes on nEuroimaging (STRIVE v1) Neuroimaging standards for research into small vessel disease and its contribution to ageing and neurodegeneration. Lancet Neurol. 2013;12(8):822–838. 23867200 10.1016/S1474-4422(13)70124-8 PMC3714437

[23] Greene-Schloesser D, Robbins ME, Peiffer AM, Shaw EG, Wheeler KT, Chan MD Radiation-induced brain injury: a review. Front Oncol. 2012;2:73. 22833841 10.3389/fonc.2012.00073 PMC3400082

[24] Sacco RL, Kasner SE, Broderick JP, et al; American Heart Association Stroke Council, Council on Cardiovascular Surgery and Anesthesia; Council on Cardiovascular Radiology and Intervention; Council on Cardiovascular and Stroke Nursing; Council on Epidemiology and Prevention; Council on Peripheral Vascular Disease; Council on Nutrition, Physical Activity and Metabolism An updated definition of stroke for the 21st century: a statement for healthcare professionals from the American Heart Association/American Stroke Association. Stroke. 2013;44(7):2064–2089. 23652265 10.1161/STR.0b013e318296aeca PMC11078537

[25] Easton JD, Saver JL, Albers GW, et al; American Heart Association; American Stroke Association Stroke Council; Council on Cardiovascular Surgery and Anesthesia; Council on Cardiovascular Radiology and Intervention; Council on Cardiovascular Nursing; Interdisciplinary Council on Peripheral Vascular Disease Definition and evaluation of transient ischemic attack: a scientific statement for healthcare professionals from the American Heart Association/American Stroke Association Stroke Council; Council on Cardiovascular Surgery and Anesthesia; Council on Cardiovascular Radiology and Intervention; Council on Cardiovascular Nursing; and the Interdisciplinary Council on Peripheral Vascular Disease. The American Academy of Neurology affirms the value of this statement as an educational tool for neurologists. Stroke. 2009;40(6):2276–2293. 19423857 10.1161/STROKEAHA.108.192218

[26] Burns TC, Awad AJ, Li MD, et al. Radiation-induced brain injury: low-hanging fruit for neuroregeneration. Neurosurg Focus. 2016;40(5):E3. 10.3171/2016.2.FOCUS161 27132524

[27] Fazekas F, Chawluk JB, Alavi A, Hurtig HI, Zimmerman RA MR signal abnormalities at 1.5 T in Alzheimer’s dementia and normal aging. AJR Am J Roentgenol. 1987;149(2):351–356. 3496763 10.2214/ajr.149.2.351

[28] U.S. Department of Health and Human Services. National Institutes of Health and National Cancer Institute. Common Terminology Criteria for Adverse Events (CTCAE), version 5.0 (2017) https://ctep.cancer.gov/protocoldevelopment/electronic_applications/docs/CTCAE_v5_Quick_Reference_8.5x11.pdf. Accessed November 27, 2017.

[29] Song Y, Manson JE, Tinker L, et al. Insulin sensitivity and insulin secretion determined by homeostasis model assessment and risk of diabetes in a multiethnic cohort of women: the Women’s Health Initiative Observational Study. Diabetes Care. 2007;30(7):1747–1752. 17468352 10.2337/dc07-0358 PMC1952235

[30] El-Fayech C, Haddy N, Allodji RS, et al. Cerebrovascular diseases in childhood cancer survivors: role of the radiation dose to Willis circle arteries. Int J Radiat Oncol Biol Phys. 2017;97(2):278–286. 28068236 10.1016/j.ijrobp.2016.10.015

[31] Miura M, Nakajima M, Fujimoto A, et al. High prevalence of small vessel disease long after cranial irradiation. J Clin Neurosci. 2017;46:129–135. 28974389 10.1016/j.jocn.2017.09.008

[32] Desai SS, Paulino AC, Mai WY, et al. Radiation-induced moyamoya syndrome. Int J Radiat Oncol Biol Phys. 2006;65(4):1222–1227. 16626890 10.1016/j.ijrobp.2006.01.038

[33] de Bruijn RF, Akoudad S, Cremers LG, et al. Determinants, MRI correlates, and prognosis of mild cognitive impairment: the Rotterdam Study. J Alzheimers Dis. 2014;42(suppl 3):S239–S249. 24825566 10.3233/JAD-132558

[34] Kaffashian S, Tzourio C, Zhu YC, Mazoyer B, Debette S Differential effect of white-matter lesions and covert brain infarcts on the risk of ischemic stroke and intracerebral hemorrhage. Stroke. 2016;47(7):1923–1925. 27283199 10.1161/STROKEAHA.116.012734

[35] Kuller LH, Longstreth WT Jr, Arnold AM, Bernick C, Bryan RN, Beauchamp NJ Jr; Cardiovascular Health Study Collaborative Research Group White matter hyperintensity on cranial magnetic resonance imaging: a predictor of stroke. Stroke. 2004;35(8):1821–1825. 15178824 10.1161/01.STR.0000132193.35955.69

[36] Jacola LM, Ashford JM, Reddick WE, et al. The relationship between working memory and cerebral white matter volume in survivors of childhood brain tumors treated with conformal radiation therapy. J Neurooncol. 2014;119(1):197–205. 24847967 10.1007/s11060-014-1476-4 PMC4133306

[37] Sipilä JOT, Posti JP, Ruuskanen JO, Rautava P, Kytö V Stroke hospitalization trends of the working-aged in Finland. PLoS One. 2018;13(8):e0201633. 30067825 10.1371/journal.pone.0201633 PMC6070270

[38] Lin DD, Filippi CG, Steever AB, Zimmerman RD Detection of intracranial hemorrhage: comparison between gradient-echo images and b(0) images obtained from diffusion-weighted echo-planar sequences. AJNR Am J Neuroradiol. 2001;22(7):1275–1281. 11498414 PMC7975215

[39] Morrison MA, Hess CP, Clarke JL, et al. Risk factors of radiotherapy-induced cerebral microbleeds and serial analysis of their size compared with white matter changes: a 7T MRI study in 113 adult patients with brain tumors. J Magn Reson Imaging. 2019;50(3):868–877. 30663150 10.1002/jmri.26651 PMC6642688

[40] Kralik SF, Mereniuk TR, Grignon L, et al. Radiation-induced cerebral microbleeds in pediatric patients with brain tumors treated with proton radiation therapy. Int J Radiat Oncol Biol Phys. 2018;102(5):1465–1471. 30092336 10.1016/j.ijrobp.2018.07.2016

[41] Taki S, Higashi K, Oguchi M, et al. Changes in regional cerebral blood flow in irradiated regions and normal brain after stereotactic radiosurgery. Ann Nucl Med. 2002;16(4):273–277. 12126097 10.1007/BF03000106

[42] Böhm M, Schumacher H, Teo KK, et al. Achieved blood pressure and cardiovascular outcomes in high-risk patients: results from ONTARGET and TRANSCEND trials. Lancet. 2017;389(10085):2226–2237. 28390695 10.1016/S0140-6736(17)30754-7

[43] Bezerra DC, Sharrett AR, Matsushita K, et al. Risk factors for lacune subtypes in the Atherosclerosis Risk in Communities (ARIC) Study. Neurology. 2012;78(2):102–108. 22170882 10.1212/WNL.0b013e31823efc42 PMC3466671

[44] Wright JM, Musini VM, Gill R First-line drugs for hypertension. Cochrane Database Syst Rev. 2018;4:CD001841. 29667175 10.1002/14651858.CD001841.pub3 PMC6513559

[45] Zonneveld TP, Richard E, Vergouwen MD, et al. Blood pressure-lowering treatment for preventing recurrent stroke, major vascular events, and dementia in patients with a history of stroke or transient ischaemic attack. Cochrane Database Syst Rev. 2018;7:CD007858. 30024023 10.1002/14651858.CD007858.pub2 PMC6513249

[46] Wang X, Dong Y, Qi X, Huang C, Hou L Cholesterol levels and risk of hemorrhagic stroke: a systematic review and meta-analysis. Stroke. 2013;44(7):1833–1839. 23704101 10.1161/STROKEAHA.113.001326

[47] Iso H, Jacobs DR Jr, Wentworth D, Neaton JD, Cohen JD Serum cholesterol levels and six-year mortality from stroke in 350,977 men screened for the multiple risk factor intervention trial. N Engl J Med. 1989;320(14):904–910. 2619783 10.1056/NEJM198904063201405

[48] Leppälä JM, Virtamo J, Fogelholm R, Albanes D, Heinonen OP Different risk factors for different stroke subtypes: association of blood pressure, cholesterol, and antioxidants. Stroke. 1999;30(12):2535–2540. 10582974 10.1161/01.str.30.12.2535

[49] Ylikoski A, Erkinjuntti T, Raininko R, Sarna S, Sulkava R, Tilvis R White matter hyperintensities on MRI in the neurologically nondiseased elderly. Analysis of cohorts of consecutive subjects aged 55 to 85 years living at home. Stroke. 1995;26(7):1171–1177. 7604409 10.1161/01.str.26.7.1171

[50] Garde E, Mortensen EL, Krabbe K, Rostrup E, Larsson HB Relation between age-related decline in intelligence and cerebral white-matter hyperintensities in healthy octogenarians: a longitudinal study. Lancet. 2000;356(9230):628–634. 10968435 10.1016/S0140-6736(00)02604-0

[51] Szerlip N, Rutter C, Ram N, et al. Factors impacting volumetric white matter changes following whole brain radiation therapy. J Neurooncol. 2011;103(1):111–119. 20725847 10.1007/s11060-010-0358-7

[52] Godin O, Tzourio C, Maillard P, Mazoyer B, Dufouil C Antihypertensive treatment and change in blood pressure are associated with the progression of white matter lesion volumes: the Three-City (3C)-Dijon Magnetic Resonance Imaging Study. Circulation. 2011;123(3):266–273. 21220733 10.1161/CIRCULATIONAHA.110.961052

[53] Tsuruda JS, Kortman KE, Bradley WG, Wheeler DC, Van Dalsem W, Bradley TP Radiation effect on cerebral white matter: MRI evaluation. Am J Roentgenol. 1987;149(1):165–171. 3495977 10.2214/ajr.149.1.165

[54] Mamlouk MD, Handwerker J, Ospina J, Hasso AN Neuroimaging findings of the post-treatment effects of radiation and chemotherapy of malignant primary glial neoplasms. Neuroradiol J. 2013;26(4):396–412. 24007728 10.1177/197140091302600405 PMC4202820

