# Supplementary material for: Radiation-induced accelerated aging of the brain vasculature in young adult survivors of childhood brain tumors
Source: Neurooncol Pract. 2020 Feb 7;7(4):415–27. doi: 10.1093/nop/npaa002 (PMC7393284; doi:10.1093/nop/npaa002)
Supplement: npaa002_suppl_Supplementary_Table_1 [file npaa002_suppl_supplementary_table_1.docx]

**SUPPLEMENTAL TABLE 1.** Tumor characteristics and tumor treatment according to cerebrovascular disease, small-vessel disease, and large-vessel disease

Cerebrovascular disease Small-vessel disease Large-vessel disease

Yes No Yes No Yes No

(*n = 44) (n = 26) (n = 27) (n = 43) (n = 13) (n = 57)*

Age at diagnosis, years; Mean (SD) 8.0 (4.5) 8.8 (4.0) 7.6 (4.6) 8.7 (4.1) 8.4 (4.7) 8.2 (4.3)

OR (95% CI) 0.96 (0.85 to 1.07) 0.94 (0.84 to 1.06) 1.01 (0.88 to 1.17)

*P*^a^ .442 .323 .867

Age at follow-up visit, years;

Mean (SD) 29.2 (6.9) 26.3 (6.1) 27.7 (6.3) 28.4 (7.1) 32.4 (6.7) 27.2 (6.4)

OR (95% CI) 1.07 (0.99 to 1.16) 0.98 (0.92 to 1.06) 1.12 (1.02 to 1.24)

*P*^a^ .086 .670 .016^b^

Tumor location, *n* (%)

Infratentorial 26 (59) 11 (42) 18 (67) 19 (44) 9 (69) 28 (49)

Supratentorial 18 (41) 15 (58) 9 (33) 24 (56) 4 (31) 29 (51)

OR for infratentorial (95% CI) 0.51 (0.19 to 1.36) 0.40 (0.15 to 1.08) 0.43 (0.12 to 1.56)

*P*^a^ .177 .070 .198

Radiation dose in Gy,

Mean (SD) 51.8 (4.9) 50.1 (5.7) 50.9 (4.0) 51.3 (5.9) 51.2 (5.4) 51.2 (5.2)

OR (95% CI) 1.07 (0.97 to 1.17) 0.98 (0.90 to 1.08) 1.00 (0.89 to 1.12)

*P*^a^ .202 .739 .995

Radiation, *n* (%)

Local 25 (57) 12 (46) 13 (48) 24 (56) 8 (62) 29 (51)

Cranial/craniospinal 19 (43) 14 (54) 14 (52) 19 (44) 5 (38) 28 (49)

OR for local (95% CI) 0.65 (0.25 to 1.73) 1.36 (0.52 to 3.57) 0.65 (0.19 to 2.22)

*P*^a^ .389 .532 .489

Radiation, *n* (%)

Local or cranial 26 (59) 14 (54) 14 (52) 26 (60) 8 (61) 32 (56)

Craniospinal 18 (41) 12 (46) 13 (48) 17 (40) 5 (39) 25 (44)

OR for local or cranial (95% CI) 1.24 (0.47 to 3.29) 0.70 (0.27 to 1.86) 1.25 (0.36 to 4.29)

*P*^a^ .669 .479 .723

Chemotherapy, *n* (%)

Yes 28 (64) 17 (65) 19 (70) 26 (60) 9 (69) 36 (63)

No 16 (36) 9 (35) 8 (30) 17 (40) 4 (31) 21 (37)

OR for chemotherapy (95% CI) 0.93 (0.34 to 2.56) 1.55 (0.56 to 4.34) 1.31 (0.36 to 4.79)

*P*^a^ .883 .401 .681

Ventriculoperitoneal shunt, *n* (%)

Yes 29 (66) 12 (46) 21 (78) 20 (47) 9 (69) 32 (63)

No 15 (34) 14 (54) 6 (22) 23 (53) 4 (31) 25 (37)

OR to ventriculoperitoneal shunt,

(95% CI) 2.26 (0.84 to 6.08) 4.03 (1.36 to 11.94) 1.76 (0.48 to 6.38)

*P^a^* .108 .012^b^ .391

^a^ Logistic regression analysis; ^b^ Significant level is 0.05
