# Supplementary material for: Radiation-induced accelerated aging of the brain vasculature in young adult survivors of childhood brain tumors
Source: Neurooncol Pract. 2020 Feb 7;7(4):415–27. doi: 10.1093/nop/npaa002 (PMC7393284; doi:10.1093/nop/npaa002)
Supplement: npaa002_suppl_Supplementary_Table_2 [file npaa002_suppl_supplementary_table_2.docx]

**SUPPLEMENTARY TABLE 2.** Tumor characteristics, tumor treatment and study MRI findings according ischemic infarct or TIA, lacunar infarct and white matter hyperintensities

Ischemic infarct Lacunar infarct White matter lesions

Yes (*n* = 6) No (*n* = 64) Yes (*n* = 7) No (*n* = 63) Yes (*n* = 34) No (*n* =36)

Age at diagnosis, years, Mean (SD) 9.8 (5.1) 8.1 (4.2) 8.3 (4.4) 8.0 (4.5) 8.5 (4.2)

OR (95% CI) 1.10 (0.90 to 1.34) 0.98 (0.82 to 1.18) 0.98 (0.88 to 1.09)

*p ^a^* .366 .835 .675

Age at follow-up visit, years,

Mean (SD) 32.3 (7.8) 27.7 (6.6) 28.2 (5.5) 28.1 (6.9) 30.6 (6.4) 25.8 (6.3)

OR (95% CI) 1.10 (0.97 to 1.25) 1.00 (0.89 to 1.12) 1.13 (1.04 to 1.23)

*p ^a^* .126 .982 .005^b^

Tumor location, *n* (%)

Supratentorial 1 (17) 32 (50) 4 (57) 29 (46) 15 (44) 18 (50)

Infratentorial 5 (83) 32 (50) 3 (43) 34 (54) 19 (55) 18 (50)

OR for supratentorial (95% CI) 0.20 (0.02 to 1.81) 1.56 (0.32 to 7.57) 0.78 (0.31 to 2.02)

*p ^a^* .152 .579 .622

Radiation dose in Gy, Mean (SD) 49.9 (2.6) 51.3 (5.4) 49.9 (4.8) 51.3 (5.3) 52.3 (4.6) 50.1 (5.7)

OR (95% CI) 0.96 (0.83 to 1.11) 0.96 (0.83 to 1.10) 1.09 (0.98 to 1.21)

*p ^a^* .546 .515 .098

Radiation, *n* (%)

Local 3 (50) 34 (53) 4 (57) 33 (51) 22 (65) 15 (42)

Whole brain with or

without spinal 3(50) 30 (47) 3 (43) 30 (49) 12 (35) 21 (58)

OR for local (95% CI) 1.13 (0.21 to 6.04) 0.83 (0.17 to 4.00) 0.39 (0.15 to 1.02)

*p ^a^* .883 .811 .056

Radiation, *n* (%)

Local or whole-brain 3 (50) 37 (58) 4 (57) 36 (57) 24 (71) 16 (44)

Craniospinal 3 (50) 27 (42) 3 (43) 27 (43) 10 (29) 20 (56)

OR for local or cranial (95% CI) 0.73 (0.14 to 3.90) 1.00 (0.21 to 4.85) 3.00 (1.12 to 8.06)

*p ^a^* .712 1.000 .029^b^

Chemotherapy, N (%)

Yes 5 (83) 40 (63) 3 (43) 42 (67) 21 (62) 24 (67)

No 1 (17) 24 (37) 4 (57) 21 (33) 13 (38) 12 (33)

OR for chemotherapy (95% CI) 3.00 (0.33 to 27.03) 0.38 (0.08 to 1.83) 0.81 (0.30 to 2.15)

*p ^a^* .329 .225 .669

Ventriculoperitoneal

shunt, *n* (%)

Yes 5 (83) 36 (56) 7 (100.0) 34 (54) 21 (62) 24 (67)

No 1 (17) 28 (44) 0 (0) 29 (46) 13 (38) 12 (33)

OR for ventriculoperitoneal shunt

(95% CI) 3.89 (0.43 to 35.21) 332597744,7 (NA) 2.09 (0.79 to 5.52)

*P ^a^* .227 .998 ^c^ .137

Focal hemosiderin

deposits, *n* (%)

Yes 2 (33) 21 (33) 4 (57) 19 (30)

No 4 (67) 43 (67) 3 (43) 44 (70)

OR (95% CI) 0.98 (0.17 to 6.05) 3.09 (0.63 to 15.15)

*P ^a^* .979 .165

Mineralizing

microangiopathy, *n* (%)

Yes 2 (33) 19 (30) 3 (43) 18 (29)

No 4 (67) 45 (70) 4 (57) 45 (71)

OR for mineralizing

microangiopathy (95%CI) 1.18 (0.20 to 7.02) 1.88 (0.38 to 9.23)

*P ^a^* .852 .439

White matter hyperintensities, *n* (%)

Yes 4 (67) 30 (47) 6 (86) 28 (44)

No 2 (33) 34 (53) 1 (14) 35 (56)

OR for white matter

hyperintensities (95%CI) 2.27 (0.39 to 13.27) 7.50 (0.85 to 65.99)

*P^a^* .364 .069

Periventricular white

matter hyperintensities, *n* (%)^d^

0 3 (50) 47 (73) 3 (43) 47 (75)

1 2 (33) 10 (16) 1 (14) 11 (18)

2 1 (17) 6 (9) 3 (43) 4 (6)

3 0 (0) 1 (2) 0 (0) 1 (1)

*P ^c^* .430 .106

Deep white matter hyperintensities,

*n* (%)^d^

0 4 (66) 41 (64) 2 (29) 43 (68)

1 1 (17) 12 (19) 3 (43) 10 (16)

2 0 (0) 9 (14) 1 (14) 8 (13)

3 1 (17) 2 (3) 1 (14) 2 (3)

*P ^c^* .409 .110

NA=not applicable, ^a^ Logistic regression analysis; ^b^ Significant level 0.05; ^c^ Chi Square Exact Test; ^d^ According to Fazekas scale
