# Supplementary material for: Radiation-induced accelerated aging of the brain vasculature in young adult survivors of childhood brain tumors
Source: Neurooncol Pract. 2020 Feb 7;7(4):415–27. doi: 10.1093/nop/npaa002 (PMC7393284; doi:10.1093/nop/npaa002)
Supplement: npaa002_suppl_Supplementary_Table_3 [file npaa002_suppl_supplementary_table_3.docx]

**SUPPLEMENTAL TABLE 3.** White matter hyperintensities according to Fazekas grade and treatment-related characteristics and atherosclerotic risk-factors

Fazekas scale

Periventricular (PVH) 0 (*n* = 50) 1 (*n* = 12) 2 (*n* = 7) 3 (*n* = 1) *P*

Deep white matter (DWMH) 0 (*n* = 45) 1 (*n* = 13) 2 (*n* = 9) 3 (*n* =3)

Radiation dose in Gy, Mean (SD)

PVH 50.7 (6.0) 51.8 (2.9) 52.7 (1.8) 54.0 .627^a^

DWMH 50.5 (5.1) 50.1 (4.7) 56.0 (5.0) 51.8 (1.9) .035^a,b,c^

Radiotherapy, *n* (%) .603^d^

PVH

Local 25 (50) 8 (67) 4 (57) 0 (0)

Whole-brain 25 (50) 4 (33) 3 (43) 1 (100)

DWMH .633^b^

Local 21 (47) 8 (62) 6 (67) 2 (67)

Whole-brain 24 (53) 5 (38) 3 (33) 1 (33)

Chemotherapy, *n* (%) PVH 33 (66) 7 (58) 4 (57) 1 (100) .856^d^

DWMH 30 (67) 8 (62) 5 (56) 2 (67) .910^d^

Ventriculoperitoneal shunt, *n* (%)

PVH 26 (52) 7 (58) 7 (100) 1 (100) .068^d^

DWMH 24 (53) 9 (69) 6 (67) 2 (67) .720^d^

Systolic blood pressure in mmHG,

Mean (SD)

PVH 127 (15)^e^ 137 (12) 141 (24) 159 .055^a^

DWMH 129 (16) 132 (13) 134 (21) 146 (30) .590^a^

Diastolic blood pressure in mmHg,

Mean (SD)

PVH 78 (10)^e^ 84 (10) 89 (17) 102 .069^a^

DWMH 78 (11) 82 (9) 86 (12) 95 (23) .195^a^

BMI in kg/m^2^, Mean (SD)

PVH 24.8 (6.6) 24.5 (3.9) 30.2 (8.2) 22.6 .410^a,b,f^

DWMH 24.4 (6.3) 24.5 (4.5) 32.0 (5.6) 21.3 (9.3) .011^a^

^a^ Kruskal-Wallis test; ^b^ Significant level 0.05, ^c^ 1<2; *P* = 0.047; 0<2; *P* = 0.036; ^d^ Chi-square exact test; ^e^ *n* = 46; ^f^ 1<2; *P* = 0.047; 0<2; *P* = 0.036
