# Supplementary material for: Radiation-induced accelerated aging of the brain vasculature in young adult survivors of childhood brain tumors
Source: Neurooncol Pract. 2020 Feb 7;7(4):415–27. doi: 10.1093/nop/npaa002 (PMC7393284; doi:10.1093/nop/npaa002)
Supplement: npaa002_suppl_Supplementary_Table_4 [file npaa002_suppl_supplementary_table_4.docx]

**SUPPLEMENTAL TABLE 4.** Association of cerebrovascular disease, markers of blood sugar metabolism and body mass index.

Triglyserides Glucose GHbA1c HOMA Body mass Waist circumference

in mol/l in mmol/l in % index in kg/m^2^ circumference in cm

Cerebrovascular disease

Yes 1.7 (1.6) 5.3 (0.7) 5.5 (0.6) 11.9 (53.0) 24.8 (6.2) 90.0 (14.6)

No 1.3 (0.8) 5.4 (0.9) 5.6 (0.8) 12.1 (43.1) 26.1 (7.0) 91.1 (16.7)

OR 1.29 0.87 0.83 1.00 0.97 1.00

(95% CI) (0.76 to 2.17) (0.44 to 1.66)(0.40 to 1.70)(0.99 to 1.01) (0.96 to 1.11)(0.97 to 1.04)

*p* .348 .646 .606 .991 .392 .783

Small-vessel disease

Yes 1.5 (0.9) 5.4 (0.7) 5.4 (0.3) 3.5 (3.0) 23.9 (5.8) 88.9 (15.1)

No 1.5 (1.6) 5.3 (0.8) 5.6 (0.8) 17.2 (62.0) 26.1 (6.8) 91.4 (15.5)

OR 1.01 1.22 0.65 0.98 0.95 0.99

(95% CI) (0.70 to 1.46) (0.62 to 2.37)(0.25 to 1.68)(0.92 to 1.05) (0.87 to 1.02)(0.96 to 1.02)

*p* .964 .564 .373 .602 .168 .506

Large-vessel disease

Yes 2.3 (2.6) 5.1 (0.7) 5.6 (1.0) 37.0 (104.3) 25.1 (7.5) 90.9 (15.6)

No 1.4 (0.8) 5.4 (0.8) 5.5 (0.6) 7.4 (29.7) 25.3 (6.3) 90.3 (15.4)

OR 1.57 0.57 1.10 1.01 1.00 1.00

(95% CI) (0.92 to 2.69) (0.18 to 1.85)(0.46 to 2.62)(0.997 to 1.02) (0.91 to 1.09)(0.96 to 1.04)

*p* .100 .352 .825 .146 .935 .905

Ischemic infarct

Yes 2.1 (0.7) 5.1 (0.5) 5.3 (0.2) 2.3 (1.1) 21.9 (5.8) 83.4 (13.5)

No 1.5 (1.4) 5.4 (0.8) 5.5 (0.7) 12.3 (49.6) 25.6 (6.5) 91.1 (15.4)

OR 1.24 0.58 0.25 0.75 0.90 0.96

(95% CI) (0.79 to 1.94) (0.10 to 3.42)(0.01 to 5.98)(0.39 to 1.43) (0.76 to 1.06)(0.90 to 1.03)

*p* .351 .543 .395 .382 .190 .239

Lacunar infarct

Yes 1.7 (0.9) 5.1 (0.5) 5.3 (0.2) 2.3 (1.1) 29.7 (5.8) 102.8 (12.8)

No 1.5 (1.4) 5.4 (0.8) 5.5 (0.7) 12.6 (50.4) 24.8 (6.4) 89.0 (15.0)

OR 1.08 0.75 0.58 1.00 1.11 1.06

(95% CI) (0.64 to 1.83) (0.19 to 2.82)(0.07 to 4.72)(0.97 to 1.03) (0.99 to 1.25)(1.00to1.11)

*p* .762 .669 .608 .768 .068 .036

PVH or DWMH

Yes 1.8 (1.8) 5.4 (0.9) 5.6 (0.9) 21.1 (69.0) 26.2 (6.2) 92.8 (12.5)

No 1.2 (0.7) 5.3 (0.5) 5.4 (0.3) 3.1 (2.6) 24.4 (6.7) 88.1 (17.4)

OR 1.75 1.37 1.58 1.10 1.05 1.02

(95% CI) (0.93 to 3.25) (0.68 to 2.75)(0.68 to 3.72)(0.92 to 1.33) (0.97 to 1.13)(0.00 to 1.05)

*P* .084 .375 .293 .289 .240 .204

Smoking Estrogen treatment Family history of stroke

Yes No Yes No Yes No

(*n* = 10) (*n* =59) (*n* = 11) (*n* = 16) (*n* = 17) (*n* = 51)

Cerebrovascular disease

Yes 4 (40) 39 (66) 6 (55) 9 (56) 12 (71) 32 (63)

No 6 (60) 20 (34) 5 (45) 7 (44) 5 (29) 19 (37)

OR 0.34 0.93 1.43

(95% CI) (0.09 to 1.35) (0.20 to 4.35) (0.43 to 4.76)

*p* .126 .930 .559

Small-vessel disease

Yes 4 (40) 22 (37) 3 (27) 2 (13) 7 (41) 20 (39)

No 6 (60) 37 (63) 8 (73) 14 (87) 10 (59) 31 (61)

OR 1.12 2.63 1.09

(95% CI) (0.28 to 4.35) (0.36 to 20.00) (0.35 to 3.33)

*P* .870 .342 .886

Large-vessel disease

Yes 1 (10) 12 (20) 1 (9) 4 (25) 4 (24) 9 (18)

No 9 (90) 47 (80) 10 (91) 12 (75) 13 (76) 42 (82)

OR 0.44 0.30 1.44

(95%CI) (0.05 to 3.77) (0.03 to 3.13) (0.38 to 5.44)

*P* .451 .315 .594

Ischemic infarct

Yes 0 (0) 6 (10) 1 (9) 1 (6) 2 (12) 4 (8)

No 10 (100) 53 (90) 10 (91) 15 (94) 15 (88) 47 (92)

OR 0.00 1.49 1.56

(95% CI) (0.00) (0.08 to 25.00) (0.26 to 9.09)

*P* .999 .783 .624

Lacunar infarct

Yes 2 (20) 5 (8) 2 (18) 0 (0) 1 (6) 6 (12)

No 8 (80) 54 (92) 9 (82) 16 (100) 16 (94) 45 (88)

OR 2.70 1.50 0.64

(95% CI) (0.44 to 16.67) (0.08 to 27.0) (0.11 to 3.84)

*P*  .279 .783 .624

PVH or DWMH

Yes 7 (70) 26 (44) 4 (36) 10 (63) 8 (47) 25 (49)

No 3 (30) 33 (56) 7 (64) 6 (37) 9 (53) 26 (51)

OR 2.96 0.67 0.64

(95% CI) (0.70 to 12.66) (0.04 to 11.9) (0.11 to 3.84)

*P* .141 .783 .624
