# Supplementary material for: Radiation-induced accelerated aging of the brain vasculature in young adult survivors of childhood brain tumors
Source: Neurooncol Pract. 2020 Feb 7;7(4):415–27. doi: 10.1093/nop/npaa002 (PMC7393284; doi:10.1093/nop/npaa002)
Supplement: npaa002_suppl_Supplementary_Table_5 [file npaa002_suppl_supplementary_table_5.docx]

**SUPPLEMENTAL TABLE 5.** Multivariate analysis of cerebrovascular disease and atherosclerotic risk-factors

Adjusted OR

(95% Confidence interval) *P**

Cerebrovascular disease

Cholesterol in mmol/l 0.12 (0.01 to 1.51) .102

Low-density lipoprotein in mmol/l 6.22 (0.69 to 56.46) .104

High-density lipoprotein in mmol/l 5.25 (0.32 to 86.68) .246

Triglyserides in mmol/l 2.09 (0.68 to 6.39) .198

Fasting glucose in mmol/l 0.94 (0.33 to 2.69) .912

HOMA index 1.00 (0.96 to 1.03) .761

GhBA1c in % 0.99 (0.16 to 6.05) .990

Systolic blood pressure in mmHg 1.06 (1.00 to 1.12) .062

Diastolic blood pressure in mmHg 0.98 (0.90 to 1.06) .584

Forward stepwise likelihood

ratio method^a^ Systolic blood pressure in mmHg 1.04 (1.01 to 1.08) .022**

Large-vessel disease

Cholesterol in mmol/l 4.15 (0.15 to 115.0) .401

Low-density lipoprotein in mmol/l 0.41 (0.02 to 7.75) .410

High-density lipoprotein 1.46 (0.04 to 55.80) .836

Triglyserides in mmol/l 0.84 (0.16 to 4.30) .832

Fasting glucose in mmol/l 0.68 (0.14 to 3.34) .633

HOMA index 1.04 (0.98 to 1.10) .260

GhBA1c in % 0.17 (0.01 to 3.24) .239

Systolic blood pressure in mmHg 1.04 (0.95 to 1.15) .394

Diastolic blood pressure in mmHg 1.04 (0.91 to 1.19) .573

Forward stepwise likelihood

ratio method^a^ Systolic blood pressure in mmhHg 1.07 (1.01 to 1.12) .012**

Forward stepwise likelihood

ratio method^b^  Systolic blood pressure in mmHg 1.07 (1.02 to 1.13) .013**

Triglyserides in mmol/l 1.54 (0.88 to 2.69) .131

Small-vessel disease

Cholesterol in mmol/l 0.80 (0.08 to 8.05) .848

Low-density lipoprotein in mmol/l 1.13 (0.14 to 8.89) .910

High-density lipoprotein in mmol/l 0.57 (0.04 to 8.50) .684

Triglyserides in mmol/l 1.54 (0.51 to 4.64) .447

Fasting glucose in mmol/l 1.83 (0.59 to 5.65) .294

HOMA index 0.91 (0.72 to 1.15) .421

GhBA1c in % 1.13 (0.19 to 6.64) .890

Systolic blood pressure in mmHg 1.01 (0.95 to 1.07) .793

Diastolic blood pressure in mmHg 1.02 (0.94 to 1.11) .625

Lacunar infarct

Cholesterol in mmol/l 0.02 (0.00 to 2047.56) .496

Low-density lipoprotein in mmol/l 8.77 (0.00 to 11523.48) .652

High-density lipoprotein in mmol/l 0.00 (0.00 to 1.16) .053

Triglyserides in mmol/l 6.09 (0.05 to 811.93) .469

Fasting glucose in mmol/l 0.29 (0.04 to 1.95) .203

HOMA index 0.93 (0.84 to 1.03) .184

GhBA1c in % 1.06 (0.03 to 42.19) .974

Systolic blood pressure in mmHg 1.17 (0.91 to 1.50) .222

Diastolic blood pressure in mmHg 1.23 (0.90 to 1.70) .199

Forward stepwise likelihood

ratio method^c^ Diastolic blood pressure in mmHg 1.11 (1.02 to 1.21) .018**

Forward stepwise likelihood

ratio method^d^ High-density lipoprotein in mmol/l 0.003 ((0.00 to 0.60) .032**

Diastolic blood pressure in mmHg 1.13 (1.02 to 1.25) .021**

Forward stepwise likelihood

ratio method^e^ High-density lipoprotein in mmol/l 0.00 (0.00 to 0.39) .032**

HOMA index 0.98 (0.94 to 1.01) .194

Diastolic blood pressure in mmHg 1.20 (1.03 to 1.39) .021**

Periventricular or deep white matter

hyperintensities

Cholesterol in mmol/l 1.51 (0.11 to 20.27) .757

Low-density lipoprotein in mmol/l 0.88 (0.09 to 9.21) .913

High-density lipoprotein in mmol/l 1.10 (0.06 to 20.93) .951

Triglyserides in mmol/l 1.38 (0.43 to 4.41) .587

Fasting glucose in mmol/l 1.45 (0.44 to 4.80) .548

HOMA index 1.04 (0.95 to 1.14) .449

GhBA1c in % 0.28 (0.04 to 1.81) .181

Systolic blood pressure in mmHg 0.98 (0.93 to 1.04) .571

Diastolic blood pressure in mmHg 1.10 (1.00 to 1.20) .046**

Forward stepwise likelihood

ratio method^c^ Diastolic blood pressure in mmHg 1.08 (1.02 to 1.14) .006**

*

** Significant level is 0.05

^a^ Variables entered on step: Systolic blood pressure

^b^ Variables entered on step: Triglyrides

^c^ Variables entered on step: Diastolic blood pressure

^d^ Variables entered on step: High-density lipoprotein

^e^ Variables entered on step: Homeostasis model assessment of insulin resistance (HOMA) index
